# Loss of Nudt15 thiopurine detoxification increases direct DNA damage in hematopoietic stem cells

**DOI:** 10.1038/s41598-023-38952-7

**Published:** 2023-07-24

**Authors:** Noriaki Yamashita, Masahiro Kawahara, Takayuki Imai, Goichi Tatsumi, Ai Asai-Nishishita, Akira Andoh

**Affiliations:** Division of Gastroenterology and Hematology, Department of Medicine, Shiga University of Medical Science, Seta-Tsukinowa, Otsu, Shiga 520-2192 Japan

**Keywords:** Cell biology, Cell death, Cell growth, Stem cells, Haematopoietic stem cells

## Abstract

Thiopurines, such as 6-mercaptopurine (6-MP), are widely used as cytotoxic agents and immunosuppressants for leukemia and autoimmune or inflammatory diseases. A nonsynonymous single nucleotide polymorphism (p.Arg139Cys; R139C) of the nucleoside diphosphate-linked moiety X-type motif 15 (*NUDT15*) gene causes the loss of thiopurine detoxification, inducing myelosuppression. To understand such hematotoxicity, we investigate the effects of NUDT15 R139C on hematopoietic stem cells (HSCs) upon thiopurine administration. Using previously established *Nudt15*^*R138C*^ knock-in mice, which mimic myelosuppression in *NUDT15*^*R139C*^ homozygous or heterozygous patients following thiopurine administration, we investigated the numerical changes of HSCs and hematopoietic progenitor cells following 6-MP administration using in vivo flowcytometry and ex vivo HSC expansion. Genes differentially expressed between *Nudt15*^+*/*+^ HSCs and *Nudt15*^*R138C/R138C*^ HSCs were identified using RNA-sequencing before the emergence of 6-MP-induced HSC-damage. Gene Ontology (GO) and Transcriptional Regulatory Relationships Unraveled by Sentence-based Text Mining (TRRUST) analyses were performed to elucidate the molecular effects of 6-MP on HSCs. In *Nudt15*^*R138C/R138C*^ mice, 6-MP induced exhaustion of HSCs faster than that of multipotent progenitors and as fast as that of myeloid-committed progenitors. Ex vivo-expanded *Nudt15*^*R138C/R138C*^ HSCs were dose- and time-dependently damaged by 6-MP. GO analysis identified the DNA damage response and cell cycle process as the most strongly influenced processes in *Nudt15*^*R138C/R138C*^ HSCs. TRRUST analysis revealed that the Trp53-regulated transcriptional regulatory network is influenced prior to HSC exhaustion in *Nudt15*^*R138C/R138C*^ HSCs. The loss of NUDT15 thiopurine detoxification enhances thiopurine-mediated DNA damage via the Trp53 networks in HSCs. Therefore, caution is required in long-term thiopurine use in patients with NUDT15 R139C in view of its adverse effects on HSCs in the form of DNA damage.

## Introduction

Thiopurines, 6-mercaptopurine (6-MP) and azathioprine (AZA), are widely used as cytotoxic agents for the management of many cancers, especially acute lymphoblastic leukemia (ALL)^1,2^, and as immunosuppressants for autoimmune or inflammatory diseases, especially inflammatory bowel disease (IBD)^3^. Despite their clinical usefulness, thiopurines frequently have serious adverse effects^4^, the most common being myelosuppression, which can force patients to discontinue treatment^5^. A nonsynonymous single nucleotide polymorphism (SNP), rs116855232 (the c.415C > T variant), which gives rise to the p.Arg139Cys (R139C), in the nucleoside diphosphate-linked moiety X-type motif 15 (*NUDT15*) gene, has recently been identified as a strong risk factor for thiopurine-induced severe leukopenia in patients with IBD and ALL ^6–9^. The allele with this SNP is present in ~ 25% of the Japanese^9^ and 20% of the East Asians, but rare in Caucasians and Africans^10^. NUDT15 is a nucleotide diphosphatase that converts thio-guanosine-5′-triphosphate, an active thiopurine metabolite that functions by being incorporated into RNA or DNA, into thio-guanosine-5′-monophosphate, an inactive thiopurine metabolite, thereby detoxifying the thiopurine. NUDT15 R139C is reported to exhibit strong cytotoxicity as a result of the almost complete loss of this enzymatic activity^10^. The number of *NUDT15*^*R139C*^ alleles present correlates with myelosuppression strength, and thiopurines cannot be administered to *NUDT15*^*R139C*^ homozygous patients with non-malignant diseases such as IBD^9^.

We have previously established knock-in mice harboring a p.Arg138Cys mutation (*Nudt15*^*R138C*^) that corresponds to *NUDT15*^*R139C*^ in humans. *Nudt15*^*R138C/R138C*^ mice exhibit myelosuppression and leukopenia, with approximately tenfold higher sensitivity to 6-MP than *Nudt15*^+*/*+^ mice; thiopurine administration is consequently lethal in *Nudt15*^*R138C/R138C*^ mice^11^. At 5 days after peritoneal administration with 5 mg/kg 6-MP, the number of hematopoietic stem cells (HSCs) in *Nudt15*^*R138C/R138C*^ mice was reduced by 70%. HSCs are generally thought to be resistant to antimetabolites that interfere with DNA synthesis and repair, such as 5-fluorouracil (5-FU) and hydroxycarbamide, owing to their own relative quiescence^12–14^. While HSCs maintain dormancy during homeostasis, their self-renewal capacity is activated in response to anticancer drugs, to rescue the injured hematopoietic system^15^. However, it is unclear what damage HSCs undergo when they are remarkably sensitive to antimetabolic cytotoxic agents due to abnormal drug metabolism. To elucidate these dynamics, we examine how the loss of Nudt15 thiopurine detoxification affects HSCs upon thiopurine exposure.

## Methods

### Mice

We used *Nudt15*^*R138C*^ knock-in mice, previously established via CRISPR/Cas9 gene editing using oligo DNA to induce the c.412C > T transition^11^. This line was maintained via mating with wild-type C57BL/6 mice. Genotype sequencing was conducted using the forward primer GGCATCTAGCCTGTAATATAGACAT, reverse primer CAGAGGTAGGTAGGCAGATCTGAG, and sequencing primer CCCGGCCTGCAGGTCTATGCCACCAGGACAATTCAG. For analysis, 8–12-week-old male and female mice were used. 6-MP (Sigma-Aldrich, St. Louis, MO) was dissolved in DMSO with each injection and administered peritoneally at 5 mg/kg once daily. A dose of 5 mg /kg in mice is equivalent to 0.4 mg/kg in an adult human^16^. The recommended initial dose of 6-MP in adult Japanese IBD patients is 30 mg/day^9^. All animal experiments were approved by the Animal Research Committee of Shiga University of Medical Science (permission number 2021-5-7) and performed under specific pathogen-free conditions at the Research Center for Animal Life Science, Shiga University of Medical Science.

### Fluorescence-activated cell sorting (FACS) analysis

Bone marrow cells were isolated from the tibia, femur, and pelvis of mice and stained with the antibodies. We used antibodies directed against CD3e [145-2C11], CD4 [GK1.5], CD8a [53-6.7], CD19 [6D5], Mac1 [M1/70], Gr-1 [RB6-8C5], B220 [RA3-6B2], Ter119 [TER-119], CD127 [A7R34], CD34 [RAM34], CD16/32 [clone 93], CD135 [A2F10], c-kit [2B8], CD48 [HM-48-1], CD150 [TC15-12F12.2], Sca-1 [D7], CD45.1 [clone A20], and CD45.2 [clone 104] (Additional File 1, Supplementary Table 1). Antibodies were purchased from Thermo Fisher Scientific (Waltham, MA) or BioLegend (San Diego, CA). Lineage markers used in analysis of hematopoietic stem and progenitor cells (HSPCs) included CD3e, CD4, CD8a, CD19, Mac1, Gr-1, B220, and Ter119. The HSPC populations have been defined elsewhere^15^ (Additional File 1, Supplementary Fig. 1): HSC (CD34^−/low^CD135^−^CD48^−^CD150^+^Lineage^−^Sca-1^+^c-Kit^+^); multipotent progenitor 1–4 (MMP1–4, CD34^+^CD135^−^CD48^−^CD150^+^Lineage^−^Sca-1^+^c-Kit^+^; CD34^+^CD135^−^CD48^+^CD150^+^Lineage^−^Sca-1^+^c-Kit^+^; CD34^+^CD135^−^CD48^+^CD150^−^Lineage^−^Sca-1^+^c-Kit^+^; CD34^+^CD135^+^CD48^+^CD150^−^Lineage^−^Sca-1^+^c-Kit^+^); common myeloid progenitor (CMP, Lineage^−^Sca-1^−^c-Kit^+^CD16/32^low^CD34^low^); granulocyte-monocyte progenitor (GMP, Lineage^−^Sca-1^−^c-Kit^+^CD16/32^+^CD34^+^); and megakaryocyte-erythrocyte progenitor (MEP, Lineage^−^Sca-1^−^c-Kit^+^CD16/32^−^CD34^−^). Flow-cytometric data acquisition and cell sorting were performed using a FACSCanto™ II or FACSAriaFusion system (BD Biosciences, San Jose, CA). The data were analyzed using FACSDiva and FlowJo (BD Biosciences).

### Ex vivo HSC expansion and Cell viability assay

CD48^−^CD150^+^CD127^−^Lineage^−^Sca-1^+^c-Kit^+^ cells were sorted as HSCs after pre-enrichment of c-Kit-positive cells using MACS (Miltenyi Biotec, Bergisch Gladbach, Germany) were directly purified onto a plate well. and expanded^17^. Briefly, 50 HSC cells were directly purified onto a fibronectin-coated plate well containing F-12 media (Nacalai Tesque, Kyoto, Japan) supplemented with 10 mM HEPES, 1 × Penicillin–Streptomycin-Glutamine (Thermo Fisher Scientific), 1 × Insulin-Transferrin-Selenium-ethanolamine (Thermo Fisher Scientific), 10 ng/ml stem cell factor (Peprotech, Cranbury, NJ), 100 ng/ml thrombopoietin (Peprotech), and 1 mg/ml polyvinyl alcohol (Sigma-Aldrich). HSCs were cultured in a humidified tissue culture 37 °C incubator with 5% CO_2_. After expansion for 28 d with half medium change every few days, 5000 cells were reseeded into a well and incubated with 0.01, 0.1, or 1 µM 6-MP. DMSO was used as a control. Cell viability was assessed using a Cell Count Reagent SF (Nacalai Tesque) according to the manufacturer’s instructions. The data were obtained using an Infinite M200 Plate Reader (Tecan, Männedorf, Switzerland).

### RNA-sequencing

Twenty-four hours after peritoneal administration of 5 mg/kg 6-MP to *Nudt15*^+*/*+^ female mice (N = 3–6) and *Nudt15*^*R138C/R138C*^ female mice (N = 2–9), HSCs were isolated and pooled as each one sample. This process was repeated 3 times and finally 3 samples each for *Nudt15*^+*/*+^ HSCs and *Nudt15*^*R138C/R138C*^ HSCs were prepared for analysis. Total RNA was extracted from the isolated HSCs using a RNeasy Micro kit (QIAGEN, Hilden, Germany), according to the manufacturer’s instructions. After checking the quality using an Agilent 2100 Bioanalyzer (Agilent, Santa Clara, CA), cDNA were synthesized using the SMART-Seq v. 4 Ultra Low Input RNA Kit for Sequencing (Takara Bio USA, San Jose, CA). The libraries were prepared using the Nextera XT DNA Library Prep Kit (Illumina, San Diego, CA), and were sequenced on a NovaSeq 6000 platform (Illumina). Transcriptome sequence data were mapped to the GRCm38 mouse genome (GCA_000001635.2), and expression levels were normalized as transcripts per kilobase million using the DRAGEN Bio-IT platform v. 3.7.5 (Illumina). Differentially expressed genes were identified at cutoffs of log_2_ fold change >|1| and *p* < 0.05, using the bioconductor R package edgeR^18^. Gene Ontology (GO) analysis were performed using Metascape^19^ (http://metascape.org). The Transcriptional Regulatory Relationships Unraveled by Sentence-based Text Mining (TRRUST) database, containing 6552 TF–target interactions for 828 mouse TFs (https://www.grnpedia.org/trrust/)^20^, was used on Metascape to examine the activated transcriptional regulatory networks.

### Statistics

ANOVA followed by Šidák tests was adopted for multiple comparison analysis using GraphPad Prism v. 9 (GraphPad, La Jolla, CA). All tests were two-sided, with *p* < 0.05 considered significant.

The number of experiments and replicates are descried in methods and figure legends.

### Ethics

All methods were performed and reported in accordance with the relevant guidelines and regulations including ARRIVE guidelines.

## Results

### 6-MP induces exhaustion of HSCs faster than that of multipotent progenitors and as fast as that of myeloid-committed progenitors in ***Nudt15***^***R138C/R138C***^ mice

**Figure 1:**
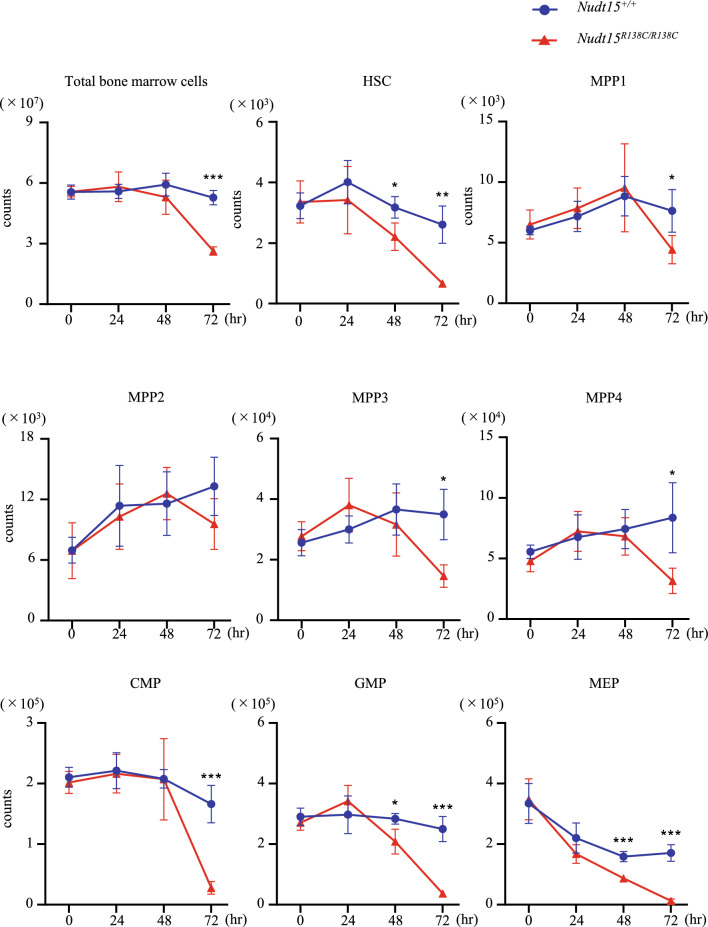
Thiopurine-induced damage of bone marrow cells by Nudt15 R138C. Bone marrow of *Nudt15*^+*/*+^ mice and *Nudt15*^*R138C/R138C*^ mice was analyzed at 0, 24, 48, and 72 h after peritoneal administration of 5 mg/kg 6-MP. Data are presented as the mean ± SD (N = 5 each condition). The experiment with one or two mice in each condition was performed independently four times. *p < 0.05, **p < 0.01, and ***p < 0.001. HSC, hematopoietic stem cell; MPP, multipotent progenitor; CMP, common myeloid progenitor; GMP, granulocyte–macrophage progenitor; MEP, megakaryocyte-erythroid progenitor.

To investigate whether the reduction of HSCs follows damage of hematopoietic progenitor cells (HPCs) or more mature blood cells in *Nudt15*^*R138C/R138C*^ mice following thiopurine administration, we counted total bone marrow cells as well as each population of HSPCs, including HSCs, MPPs, CMPs, GMPs, MEPs, in mouse bone marrow every day after 6-MP administration. The total number of bone marrow cells was significantly reduced in *Nudt15*^*R138C/R138C*^ mice, to approximately half of that in *Nudt15*^+*/*+^ mice at 72 h, but negligibly at 48 h, after 6-MP administration (Fig. 1). Numbers of myeloid-committed HPCs, such as GMPs and MEPs, were significantly reduced to approximately 70% at 48 h, and < 20% at 72 h after 6-MP administration. The number of CMPs, earlier step in myeloid-committed HPCs than GMPs and MEPs, was significantly reduced, to < 20% at 72 h, but not at all at 48 h, after 6-MP administration. The number of MPP1, MPP3, and MPP4, which are earlier steps than CMPs, GMPs, and MEPs was reduced by 40–70% at 72 h, but not at all at 48 h, after 6-MP administration. These data suggest that GMPs and MEPs are the most damaged by 6-MP, followed by reductions in the more mature blood cells that they supply, as well as in the CMPs and MPPs that supply them. By contrast, the number of HSCs was significantly reduced to 70% at 48 h and 20% at 72 h after 6-MP administration. This earlier reduction in HSCs than in MPPs after 6-MP administration in *Nudt15*^*R138C/R138C*^ mice suggests that increased 6-MP intracellular toxicity may directly affect HSCs prior to the injury-induced reduction in differentiated blood cells.

### Ex vivo expansion of ***Nudt15***^***R138C/R138C***^ HSCs is dose- and time-dependently damaged by 6-MP

**Figure 2:**
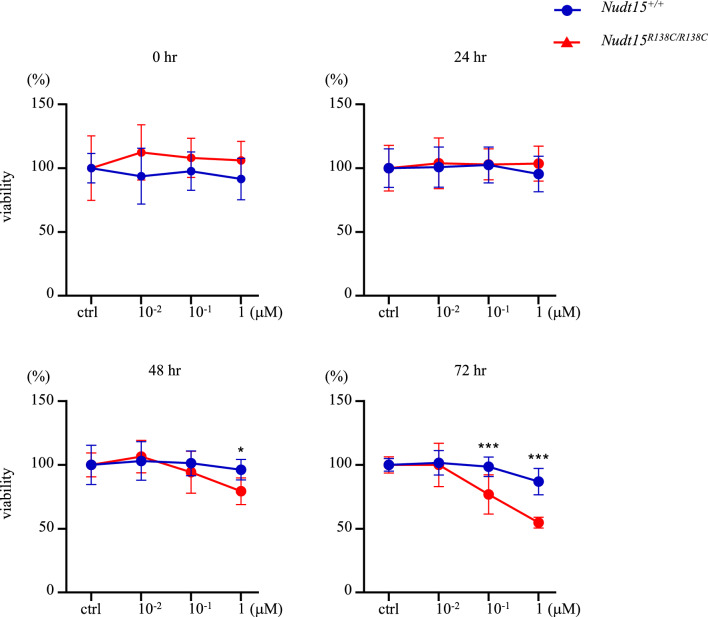
Thiopurine-induced damage of *Nudt15*^*R138C/R138C*^ ex vivo-expanded HSCs. The ex vivo-expanded HSCs were incubated with DMSO (control) or 0.01, 0.1, or 1 µM 6-MP and analyzed using the cell viability assay. Viability (%) is relative to that in the control. This experiment was performed independently twice with quadruplicates. Data are presented as the mean ± SD. *p < 0.05, **p < 0.01, and ***p < 0.001.

To investigate the direct effects of thiopurine on *Nudt15*^*R138C/R138C*^ HSCs, we used the ex vivo HSC expansion. CD48^−^CD150^+^CD127^−^Lineage^−^Sca-1^+^c-Kit^+^ cells were sorted and expanded as HSCs from *Nudt15*^*R138C/R138C*^ or *Nudt15*^+*/*+^ mice (Additional File 1, Supplementary Fig. 2). The ex vivo HSCs were verified to comparably engraft in mice and reproduce blood cells (Additional File 1, Supplementary Fig. 3). Using these cells, we performed a cell viability assay. *Nudt15*^*R138C/R138C*^ ex vivo HSCs exhibited significantly lower viability after 48 h and 72 h of culture with 1 μM 6-MP and after 72 h culture with 0.1 μM 6-MP relative to *Nudt15*^+*/*+^ HSCs (Fig. 2). This provides evidence that enhanced 6-MP intracellular toxicity by Nudt15 R138C may be linked to direct damage of HSCs.

### DNA damage response and cell cycle process are affected in ***Nudt15***^***R138C/R138C***^ HSCs prior to HSC exhaustion

**Figure 3:**
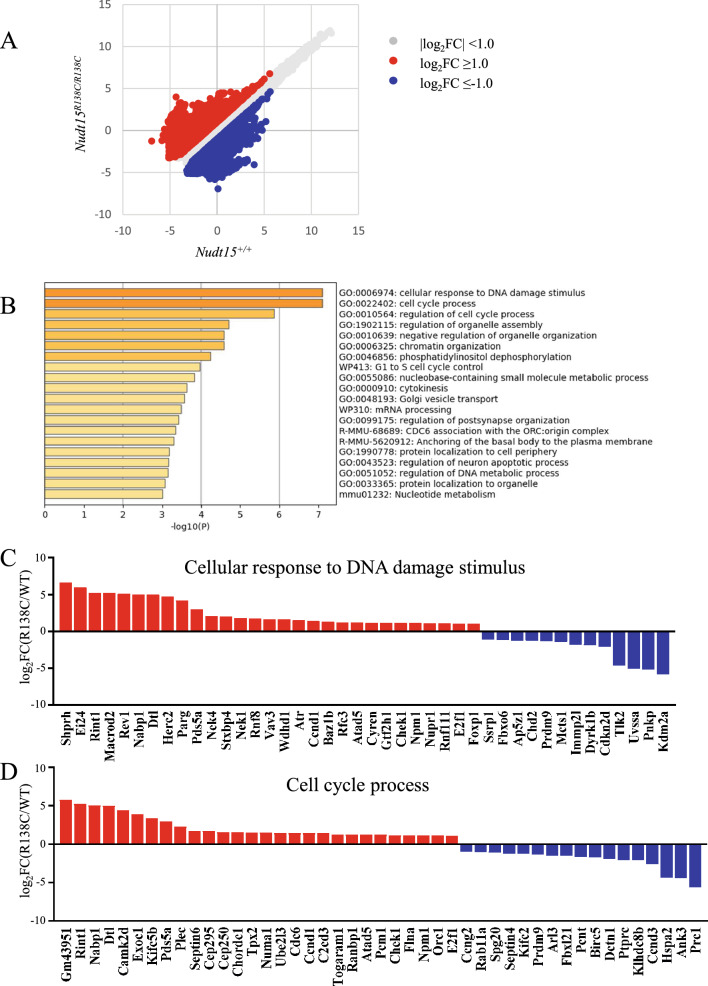
Gene expression profiling via RNA-seq and GO analyses. HSCs were sorted 24 h after peritoneal administration of 5 mg/kg 6-MP and were analyzed via RNA-seq. (**A**) Scatter plot comparing *Nudt15*^+*/*+^ HSC (WT, N = 3) and *Nudt15*^*R138C/R138C*^ HSC (R138C, N = 3). (**B**) GO analysis. Differentially expressed genes (log_2_FC >|1|, *p* < 0.05) were analyzed using Metascape (http://metascape.org). The top 20 GO terms are presented. (**C**) Change in expression of genes listed under cellular response to DNA damage stimulus. (**D**) Change in expression of genes listed under the cell cycle process.

To understand the molecular mechanisms that occur before the reduction in the number of *Nudt15*^*R138C/R138C*^ HSCs following 6-MP administration, we compared the gene expression profiles in HSCs directly isolated from *Nudt15*^*R138C/R138C*^ and *Nudt15*^+*/*+^ mice 24 h after 6-MP administration (Fig. 3A): 289 genes were upregulated and 235 genes were downregulated in *Nudt15*^*R138C/R138C*^ HSCs (Additional File 1, Supplementary Table 2). GO analysis revealed that these genes are enriched in cellular response to DNA damage and in the cell cycle process (Fig. 3B). DNA repair genes that protect against mutagenesis and chromosomal instability, such as *Shprh*^21^ and *Macrod2*^22^, were upregulated, whereas those that support cell survival, cancer development, and resistance to DNA damage agents, such as *Kdm2a*^23,24^, *Tlk2*^25^, and *Uvssa*^26^, were downregulated (Fig. 3C). Among those genes involving cell cycle process, *Cdc6* and *Orc1*^27^, which participate in initiating DNA replication, and *Ccnd1* and *E2f1*^28,29^, which have important roles in G1/S transition and DNA repair and apoptosis, were upregulated. *Cdkn2*, which induces G1 phase arrest, was downregulated^30^. *Chk1*, a key regulator of checkpoint signaling in both the unperturbed cell cycle and DNA damage response^31^, was upregulated (Fig. 3D). These results suggest that the DNA repair system and the cell cycle regulation system are closely related and simultaneously altered in HSCs prior to the reduction in the number of HPCs.

### Trp53-regulated transcriptional regulatory network is affected in ***Nudt15***^***R138C/R138C***^ HSCs before emergence of HSC exhaustion

**Figure 4:**
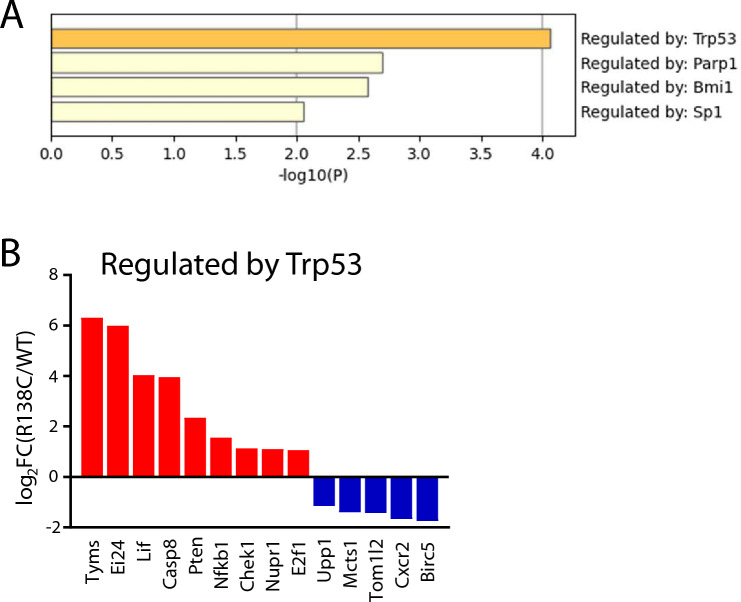
TRRUST analysis. Differentially expressed genes (log_2_FC >|1|, *p* < 0.05) analyzed using TRRUST (https://www.grnpedia.org/trrust/). (**A**) Four transcriptional regulatory networks from the list of differentially expressed genes are listed. (**B**) Change in expression of genes listed in the Trp53-regulated regulatory network.

TRRUST analysis^20^ revealed that the Trp53-regulated transcriptional regulatory network was the most highly activated (Fig. 4A). Among the genes in the Trp53 network, *Pten* and *Lif,* which maintain HSC function^32–34^, were upregulated (Fig. 4B). This is consistent with the enrichment of phosphatidylinositol dephosphorylation, revealed via GO analysis. *Ei24* and *Casp8*, involved in apoptosis^35,36^, were upregulated, suggesting that excessive DNA damage accumulates in HSCs. These results suggest that the loss of thiopurine detoxification due to Nudt15 R138C could activate the Trp53-regulated transcriptional network, inducing cell-cycle dysregulation and increasing DNA damage response, thus impairing the HSCs following 6-MP exposure.

## Discussion

We have previously demonstrated both significant bone marrow suppression and a reduction in the number of HSCs following 5 days of 6-MP administration in *Nudt15*^*R138C/R138C*^ mice. Here, we examined further how the loss of Nudt15 thiopurine detoxification affects HSCs upon thiopurine exposure. Our findings demonstrate that the loss of Nudt15 thiopurine detoxification impairs HSCs faster than or as fast as HPCs. Cells with greater proliferative capacity typically suffer greater DNA damage from antimetabolites that interfere with DNA synthesis and repair^12–14^. The steady state proliferative activity differs significantly different between HSCs and HPCs^15^. However, our findings show that HSC impairment coincides with that of myeloid-committed progenitor cells following 6-MP exposure and occurs earlier than that of MPPs in *Nudt15*^*R138C/R138C*^ mice. According to previous reports with 5-FU treatment which is another antimetabolite, dormant HSCs were induced to exit their homeostatic quiescent status from the second day after treatment and to rapidly increase BrdU incorporation over a period of 24 h^15^. HSCs are reported to undergo DNA damage as a direct consequence of their exit from dormancy^37^. Our GO analysis revealed that abnormal thiopurine metabolism facilitates the induction of the molecular mechanism for cell cycle activation by upregulating positive regulators such as *Ccnd1* and downregulating negative regulators such as *Cdkn2d* 24 h after 6-MP treatment in *Nudt15*^*R138C/R138C*^ HSCs. Simultaneously, the molecular mechanisms for cellular response to DNA damage stimulus and chromatin organization were enhanced. These molecular mechanisms are activated prior to actual HPC damage, suggesting that HSC impairment due to the loss of Nudt15 thiopurine detoxification is directly induced as an intracellular abnormality rather than as a reactive event following bone marrow injury.

Among the top upregulated genes in the GO signature of cellular response to DNA damage stimulus, *Shprh* is reported to act in a DNA damage-specific manner and to prevent mutation^21^. *Macrod2* is reported to participate in chromosomal stability and to be essential in the catalytic activity of Parp1, of which the network seems to be affected according to our TRRUST analysis^22^. Among the top downregulated genes, *Kdm2a* participates in cell survival or immortality^23,24^. *Uvssa*-knockout cells are sensitive to UV and cisplatin^26^. *TLK2* inhibition suppresses the growth of several cancers^38,39^. These findings suggest that HSCs with the loss of Nudt15 thiopurine detoxification are preparing for cell death while vigorously defending themselves against mutagenesis by thiopurine.

Our TRRUST analysis revealed activation of the Trp53-regulated transcriptional regulatory network as an initial response to 6-MP in *Nudt15*^*R138C/R138C*^ HSCs. Trp53 plays a central role in cell-cycle activation and DNA damage response^40–42^. DNA double-strand breaks activate the p53 pathway and DNA damage response in HSPCs^43^. *Lif* expression is upregulated during stress hematopoiesis and promotes maintenance of HSC self-renewal via JAK/STAT3^44,45^. *Pten*, which negatively regulates PI3/AKT/mTOR signaling^46^, is important in regulating the cell cycle and DNA damage repair^47^ and is essential for maintaining HSCs^32^. The upregulation of *Lif* and *Pten* expression may protect HSCs from delayed thiopurine metabolism. However, the simultaneous upregulation of *Ei24*, *Casp8*, and *Chk1* expression, in the background of the cytoprotective program, seems to reflect preparation for triggering cell death.

Although HSCs with the loss of Nudt15 thiopurine detoxification immediately launch defenses against DNA damage upon thiopurine exposure, the number of HSCs significantly decreases after 48 h. DNA damage due to cancer therapies has been reported to initiate clonal hematopoiesis that is related to the development of hematological malignancies and cardiovascular diseases^48,49^. Young IBD patients exposed to thiopurine have significantly higher levels of mosaic chromosomal alterations shaping a part of clonal hematopoiesis^50^. Future studies will be required to understand whether the loss of NUDT15 thiopurine detoxification could give rise to DNA damage-tolerant clones that outcompete other clones, leading to clonal hematopoiesis.

## Conclusion

The loss of NUDT15 thiopurine detoxification enhances thiopurine-mediated DNA damage via the Trp53 networks in HSCs, leading to HSC exhaustion. Although low doses of thiopurine may attenuate acute myelosuppression in *NUDT15*^*R139C*^ homozygous or heterozygous patients, long-term thiopurine use should be applied with caution, in view of its adverse effects on HSC dynamics due to DNA damage accumulation.

## Supplementary Information

Supplementary Information 1.

Supplementary Information 2.

## Data Availability

All data generated or analyzed during this study are included in this published article and its supplementary information files. The datasets generated and analyzed during the current study are available in the Gene Expression Omnibus repository, accession number GSE214604.
