# Supplementary material for: Loss of Nudt15 thiopurine detoxification increases direct DNA damage in hematopoietic stem cells
Source: Sci Rep. 2023 Jul 24;13:11908. doi: 10.1038/s41598-023-38952-7 (PMC10366091; doi:10.1038/s41598-023-38952-7)
Supplement: Supplementary file 1 — Supplementary Information 1. [file 41598_2023_38952_MOESM1_ESM.docx]

**Loss of Nudt15 thiopurine detoxification increases direct DNA damage in hematopoietic stem cells**

Noriaki Yamashita, Masahiro Kawahara*, Takayuki Imai, Goichi Tatsumi, Ai Asai-Nishishita, and Akira Andoh

Division of Gastroenterology and Hematology, Department of Medicine, Shiga University of Medical Science, Shiga, Japan

*Corresponding authors: Masahiro Kawahara

Division of Gastroenterology and Hematology, Department of Medicine, Shiga University of Medical Science, Seta-Tsukinowa, Otsu, Shiga 520-2192, Japan

Tel: +81-77-548-2217

Fax: +81-77-548-2219

E-mail: [mkawahar@belle.shiga-med.ac.jp](mailto:mkawahar@belle.shiga-med.ac.jp)

**Keywords:**

Nudt15, thiopurine, hematopoietic stem cell, DNA damage, immunosuppressant, myelosuppression

**Supplementary Information**

**Supplementary methods**

**Transplantation of ex vivo-expanded hematopoietic stem cells (HSCs)**

CD48^−^CD150^+^CD127^−^Lineage^−^Sca-1^+^c-Kit^+^ [16] cells were sorted as HSCs and expanded for four weeks. One million ex vivo expanded CD45.2 HSCs were transplanted into CD45.1 recipient mice after 6 Gy irradiation. Eight weeks from transplantation, peripheral blood chimerism was analyzed using CD45.1 [clone A20] and CD45.2 [clone 104] antibodies.

**Supplementary Figure 1**

Analysis gates for hematopoietic stem and progenitor cell (HSPC) populations. Lineage^−^Sca-1^−^c-Kit^+^, LK; Lineage^−^Sca-1^+^c-Kit^+^, LSK; hematopoietic stem cell (HSC, CD34^−/low^CD135^−^CD48^−^CD150^+^Lineage^−^Sca-1^+^c-Kit^+^); multipotent progenitor 1–4 (MPP1–4, CD34^+^CD135^−^CD48^−^CD150^+^Lineage^−^Sca-1^+^c-Kit^+^; CD34^+^CD135^−^CD48^+^CD150^+^Lineage^−^Sca-1^+^c-Kit^+^; CD34^+^CD135^−^CD48^+^CD150^−^Lineage^−^Sca-1^+^c-Kit^+^; CD34^+^CD135^+^CD48^+^CD150^−^Lineage^−^Sca-1^+^c-Kit^+^, respectively); common myeloid progenitor (CMP, Lineage^−^Sca-1^−^c-Kit^+^CD16/32^low^CD34^low^); granulocyte-monocyte progenitor (GMP, Lineage^−^Sca-1^−^c-Kit^+^CD16/32^+^CD34^+^); megakaryocyte-erythrocyte progenitor (MEP, Lineage^−^Sca-1^−^c-Kit^+^CD16/32^−^CD34^−^).

**Supplementary Figure 2**

Sorting gates for ex vivo HSC expansion. CD127 is included in the lineage. HSC, CD48^−^CD150^+^CD127^−^Lineage^−^Sca-1^+^c-Kit^+^.

**Supplementary Figure 3**

Peripheral blood chimerism from mice receiving ex vivo-expanded HSCs. CD45.1 is present on the recipient blood cells and CD45.2 is present on the donor blood cells.

**Supplementary Table 1. Antibodies.**

| Marker | Clone | Color | Company |
| --- | --- | --- | --- |
| CD3e | [145-2C11] | PE/Cy5 | BioLegend |
| CD4 | [GK1.5] | PE/Cy5 | BioLegend |
| CD8a | [53-6.7] | PE/Cy5 | BioLegend |
| CD19 | [6D5] | PE/Cy5 | Thermo Fisher Science |
| Mac1 | [M1/70] | PE/Cy5 | BioLegend |
| Gr-1 | [RB6-8C5] | PE/Cy5 | BioLegend |
| B220 | [RA3-6B2] | PE/Cy5 | BioLegend |
| Ter119 | [TER-119] | PE/Cy5 | BioLegend |
| CD127 | [A7R34] | PE/Cy5 | BioLegend |
| CD34 | [RAM34] | FITC | Thermo Fisher Science |
| CD16/32 | [93] | BV510 | BioLegend |
| CD135 | [A2F10] | Biotin | BioLegend |
| Streptavidin |  | PE/Cy7 | BioLegend |
| c-kit | [2B8] | APC | BioLegend |
| CD48 | [HM-48-1] | APC/Fire750 | BioLegend |
| CD150 | [TC15-12F12.2] | PE | BioLegend |
| Sca-1 | [D7] | BV421 | BioLegend |
| CD45.1 | [A20] | PE | BioLegend |
| CD45.2 | [104] | APC | BioLegend |

**Supplementary Table 2.** **Upregulated genes in *Nudt15^R138C/R138C^* HSCs.**

| Gene list | | Log2fold change | |  | | Gene list | | Log2fold change | |  | | Gene list | | Log2fold change | |
| --- | --- | --- | --- | --- | --- | --- | --- | --- | --- | --- | --- | --- | --- | --- | --- |
| *Mthfd1* | | 8.34 | |  | | *Aktip* | | 3.99 | |  | | *Hnrnpk* | | 2.08 | |
| *Pim1* | | 7.24 | |  | | *Klc2* | | 3.97 | |  | | *Dus1l* | | 2.06 | |
| *Prrc2c* | | 6.79 | |  | | *Casp8* | | 3.96 | |  | | *C77080* | | 2.06 | |
| *Tiam1* | | 6.68 | |  | | *Exoc1* | | 3.88 | |  | | *Pfkfb2* | | 2.04 | |
| *Cript* | | 6.66 | |  | | *Gm14434* | | 3.85 | |  | | *Papola* | | 2.01 | |
| *Pla2g6* | | 6.63 | |  | | *Ttc7b* | | 3.75 | |  | | *Rxrb* | | 2.00 | |
| *Efr3b* | | 6.62 | |  | | *Myo5b* | | 3.75 | |  | | *Ppp6r2* | | 2.00 | |
| *Shprh* | | 6.61 | |  | | *Pabpc4* | | 3.72 | |  | | *Stxbp4* | | 1.99 | |
| *Dst* | | 6.56 | |  | | *Zmynd11* | | 3.71 | |  | | *Adpgk* | | 1.98 | |
| *Psmd8* | | 6.42 | |  | | *Phf6* | | 3.70 | |  | | *Pofut2* | | 1.97 | |
| *Vps54* | | 6.31 | |  | | *Pik3c2a* | | 3.70 | |  | | *Dazap1* | | 1.97 | |
| *Tyms* | | 6.31 | |  | | *Arl15* | | 3.62 | |  | | *Mgat4a* | | 1.97 | |
| *Large2* | | 6.15 | |  | | *Agpat3* | | 3.51 | |  | | *Irf4* | | 1.97 | |
| *Ei24* | | 5.99 | |  | | *Kifc5b* | | 3.37 | |  | | *Gm3500* | | 1.94 | |
| *Lrtm2* | | 5.91 | |  | | *Naip2* | | 3.33 | |  | | *Mphosph9* | | 1.93 | |
| *Nol8* | | 5.85 | |  | | *Gtdc1* | | 2.97 | |  | | *Cep97* | | 1.93 | |
| *Gm43951* | | 5.74 | |  | | *Pds5a* | | 2.95 | |  | | *Ncor2* | | 1.93 | |
| *Arhgap21* | | 5.69 | |  | | *Cadps2* | | 2.89 | |  | | *Marveld2* | | 1.92 | |
| *Gabpb2* | | 5.63 | |  | | *Ints10* | | 2.89 | |  | | *Rnf141* | | 1.89 | |
| *Shank3* | | 5.53 | |  | | *Prr14* | | 2.84 | |  | | *Krba1* | | 1.89 | |
| *Sh3tc1* | | 5.49 | |  | | *Vps13a* | | 2.82 | |  | | *Cpsf1* | | 1.89 | |
| *Tsen34* | | 5.38 | |  | | *Rnf121* | | 2.74 | |  | | *Rtn3* | | 1.88 | |
| *Prdm4* | | 5.33 | |  | | *Prdm11* | | 2.74 | |  | | *Pogk* | | 1.85 | |
| *Eif4g1* | | 5.26 | |  | | *Rnf144a* | | 2.71 | |  | | *Smarcc1* | | 1.83 | |
| *Rbm4b* | | 5.22 | |  | | *Atg16l1* | | 2.68 | |  | | *Nek1* | | 1.82 | |
| *Rint1* | | 5.22 | |  | | *Ccpg1* | | 2.66 | |  | | *Exosc10* | | 1.81 | |
| *Cd200r4* | | 5.19 | |  | | *Ntpcr* | | 2.65 | |  | | *Sema4a* | | 1.79 | |
| *Macrod2* | | 5.19 | |  | | *Zdhhc3* | | 2.61 | |  | | *Abcb8* | | 1.78 | |
| *Zfp114* | | 5.15 | |  | | *Disp1* | | 2.53 | |  | | *Golim4* | | 1.78 | |
| *Ttc19* | | 5.14 | |  | | *Cdh23* | | 2.47 | |  | | *Lrfn1* | | 1.77 | |
| *Dcaf11* | | 5.09 | |  | | *Nqo2* | | 2.46 | |  | | *Pum3* | | 1.77 | |
| *Rev1* | | 5.08 | |  | | *Pqlc2* | | 2.45 | |  | | *Furin* | | 1.75 | |
| *Nabp1* | | 5.01 | |  | | *Ano8* | | 2.41 | |  | | *Baz2a* | | 1.74 | |
| *Dtl* | | 4.98 | |  | | *Tspan5* | | 2.38 | |  | | *Arrb2* | | 1.73 | |
| *Chn1* | | 4.94 | |  | | *Rgs12* | | 2.38 | |  | | *Rnf8* | | 1.73 | |
| *Cept1* | | 4.81 | |  | | *Pten* | | 2.35 | |  | | *Insyn2b* | | 1.72 | |
| *Herc2* | | 4.72 | |  | | *Zfp142* | | 2.34 | |  | | *Synj2* | | 1.72 | |
| *Slc25a53* | | 4.68 | |  | | *Pan2* | | 2.33 | |  | | *Nlrc5* | | 1.72 | |
| *Mef2d* | | 4.66 | |  | | *Atxn7* | | 2.32 | |  | | *Pard3b* | | 1.72 | |
| *Kcnrg* | | 4.65 | |  | | *Plec* | | 2.27 | |  | | *Bop1* | | 1.71 | |
| *Adgrg1* | | 4.64 | |  | | *Dlg2* | | 2.23 | |  | | *Nudt5* | | 1.71 | |
| *9430015G10Rik* | | 4.49 | |  | | *Wars* | | 2.20 | |  | | *Sipa1l3* | | 1.71 | |
| *Inpp4b* | | 4.40 | |  | | *Pofut1* | | 2.15 | |  | | *Septin6* | | 1.70 | |
| *Camk2d* | | 4.40 | |  | | *Rpap2* | | 2.15 | |  | | *Orc3* | | 1.69 | |
| *Shroom2* | | 4.22 | |  | | *Usp9x* | | 2.14 | |  | | *Cep295* | | 1.69 | |
| *Parg* | | 4.19 | |  | | *Vps50* | | 2.14 | |  | | *Kif24* | | 1.67 | |
| *Plpp7* | | 4.17 | |  | | *Baiap2* | | 2.13 | |  | | *Pum2* | | 1.67 | |
| *Fstl3* | | 4.16 | |  | | *Diaph2* | | 2.12 | |  | | *Zbtb25* | | 1.67 | |
| *Slc25a25* | | 4.03 | |  | | *Nek4* | | 2.11 | |  | | *Trps1* | | 1.67 | |
| *Lif* | | 4.03 | |  | | *Dnaja1* | | 2.10 | |  | | *Prrc2b* | | 1.66 | |
| *Rcbtb1* | | 4.00 | |  | | *Taf1* | | 2.09 | |  | | *Rai14* | | 1.65 | |
| Gene list | Log2fold change | |  | | Gene list | | Log2fold change | |  | | Gene list | | Log2fold change | |  |
| *Vav3* | 1.64 | |  | | *Slc25a44* | | 1.33 | |  | | *Orc1* | | 1.11 | |  |
| *Wdhd1* | 1.64 | |  | | *Ubtf* | | 1.31 | |  | | *Ttc17* | | 1.10 | |  |
| *Ripk3* | 1.63 | |  | | *Crybg3* | | 1.31 | |  | | *Kmt2a* | | 1.10 | |  |
| *Selenoi* | 1.63 | |  | | *Baz1b* | | 1.31 | |  | | *Nupr1* | | 1.09 | |  |
| *Chd7* | 1.60 | |  | | *Zfp120* | | 1.30 | |  | | *Srcap* | | 1.09 | |  |
| *Cd209g* | 1.59 | |  | | *Brpf1* | | 1.30 | |  | | *Atp6ap1* | | 1.09 | |  |
| *Dnm2* | 1.59 | |  | | *Slc9a9* | | 1.29 | |  | | *Chtf8* | | 1.09 | |  |
| *Nfkb1* | 1.56 | |  | | *Mga* | | 1.29 | |  | | *Slc25a13* | | 1.09 | |  |
| *Arnt* | 1.56 | |  | | *Cmpk2* | | 1.29 | |  | | *Sec31a* | | 1.08 | |  |
| *Cep250* | 1.54 | |  | | *Pkp4* | | 1.28 | |  | | *Uros* | | 1.08 | |  |
| *Sacs* | 1.54 | |  | | *Foxp4* | | 1.28 | |  | | *Pcnx* | | 1.07 | |  |
| *Chordc1* | 1.54 | |  | | *Pkmyt1* | | 1.28 | |  | | *Acy1* | | 1.07 | |  |
| *Zfp740* | 1.53 | |  | | *Xylb* | | 1.28 | |  | | *Rnf111* | | 1.07 | |  |
| *Sdr39u1* | 1.53 | |  | | *Pigt* | | 1.27 | |  | | *E2f1* | | 1.06 | |  |
| *Chid1* | 1.52 | |  | | *Mthfd2* | | 1.27 | |  | | *Ly9* | | 1.06 | |  |
| *Chac1* | 1.52 | |  | | *Ap5m1* | | 1.26 | |  | | *Set* | | 1.06 | |  |
| *Slc19a1* | 1.52 | |  | | *Stard13* | | 1.25 | |  | | *Zfp169* | | 1.05 | |  |
| *Hnrnph2* | 1.52 | |  | | *Fuz* | | 1.25 | |  | | *Gstcd* | | 1.05 | |  |
| *Tpx2* | 1.51 | |  | | *Nipsnap1* | | 1.25 | |  | | *Zfx* | | 1.05 | |  |
| *Numa1* | 1.51 | |  | | *Nfya* | | 1.24 | |  | | *Hyou1* | | 1.05 | |  |
| *Pfkp* | 1.51 | |  | | *Abi2* | | 1.23 | |  | | *Zfp710* | | 1.04 | |  |
| *Atr* | 1.51 | |  | | *Rfc3* | | 1.23 | |  | | *Atp11c* | | 1.04 | |  |
| *Guf1* | 1.51 | |  | | *Togaram1* | | 1.23 | |  | | *Atp11a* | | 1.03 | |  |
| *Cd151* | 1.50 | |  | | *Srek1* | | 1.23 | |  | | *2610318N02Rik* | | 1.01 | |  |
| *Mdn1* | 1.50 | |  | | *Zfp827* | | 1.22 | |  | | *Tor1aip2* | | 1.01 | |  |
| *Baz1a* | 1.49 | |  | | *Fbxo34* | | 1.21 | |  | | *Pi4kb* | | 1.01 | |  |
| *Mon2* | 1.48 | |  | | *Ranbp1* | | 1.21 | |  | | *Zfp275* | | 1.01 | |  |
| *Rnf17* | 1.46 | |  | | *Atad5* | | 1.20 | |  | | *Cwf19l1* | | 1.00 | |  |
| *Epb41l3* | 1.46 | |  | | *Trim25* | | 1.20 | |  | | *Macf1* | | 1.00 | |  |
| *Mtmr3* | 1.46 | |  | | *Slc9a8* | | 1.20 | |  | | *Foxp1* | | 1.00 | |  |
| *Gabbr1* | 1.46 | |  | | *Pcm1* | | 1.19 | |  | |  |  |  |  |  |
| *Acot4* | 1.45 | |  | | *Stau2* | | 1.19 | |  | |  |  |  |  |  |
| *Asph* | 1.44 | |  | | *Srebf2* | | 1.19 | |  | |  |  |  |  |  |
| *Pigu* | 1.43 | |  | | *Rcor3* | | 1.18 | |  | |  |  |  |  |  |
| *Ube2l3* | 1.42 | |  | | *Ecpas* | | 1.17 | |  | |  |  |  |  |  |
| *Cdc6* | 1.42 | |  | | *Mcfd2* | | 1.17 | |  | |  |  |  |  |  |
| *Ganc* | 1.41 | |  | | *Tjap1* | | 1.17 | |  | |  |  |  |  |  |
| *Ccnd1* | 1.41 | |  | | *Hdac4* | | 1.16 | |  | |  |  |  |  |  |
| *C2cd3* | 1.40 | |  | | *Gm10778* | | 1.16 | |  | |  |  |  |  |  |
| *Fyn* | 1.40 | |  | | *Sec63* | | 1.16 | |  | |  |  |  |  |  |
| *Cxadr* | 1.40 | |  | | *Kbtbd8* | | 1.15 | |  | |  |  |  |  |  |
| *Slc1a4* | 1.39 | |  | | *Cyren* | | 1.14 | |  | |  |  |  |  |  |
| *Mdm1* | 1.38 | |  | | *Gtf2h1* | | 1.14 | |  | |  |  |  |  |  |
| *Dhx9* | 1.37 | |  | | *Man1a* | | 1.14 | |  | |  |  |  |  |  |
| *Dtd1* | 1.37 | |  | | *Ipo4* | | 1.14 | |  | |  |  |  |  |  |
| *Cnnm1* | 1.37 | |  | | *Mpl* | | 1.14 | |  | |  |  |  |  |  |
| *Arhgef18* | 1.36 | |  | | *Chek1* | | 1.13 | |  | |  |  |  |  |  |
| *Fbl* | 1.36 | |  | | *Flna* | | 1.13 | |  | |  |  |  |  |  |
| *Atad1* | 1.36 | |  | | *Npm1* | | 1.13 | |  | |  |  |  |  |  |
| *Tti1* | 1.35 | |  | | *Pycr1* | | 1.13 | |  | |  |  |  |  |  |
| *Atp5c1* | 1.34 | |  | | *Hnrnpr* | | 1.12 | |  | |  |  |  |  |  |
| *Cntrl* | 1.34 | |  | | *Lipa* | | 1.12 | |  | |  |  |  |  |  |
| *Zfp445* | 1.34 | |  | | *Miga1* | | 1.11 | |  | |  |  |  |  |  |

**Supplementary Table 3.** **Downregulated genes in *Nudt15^R138C/R138C^* HSCs.**

| Gene list | Log2fold change |  | Gene list | Log2fold change |  | Gene list | Log2fold change |
| --- | --- | --- | --- | --- | --- | --- | --- |
| *Eif2d* | -1.00 |  | *Dhrs3* | -1.20 |  | *St7* | -1.48 |
| *Zkscan7* | -1.00 |  | *Sh3bp2* | -1.21 |  | *Arl3* | -1.49 |
| *Ccng2* | -1.00 |  | *Irf2* | -1.22 |  | *Zfp961* | -1.49 |
| *Mia2* | -1.00 |  | *Gatb* | -1.22 |  | *Aldh1a7* | -1.50 |
| *Thoc7* | -1.01 |  | *Plekha1* | -1.23 |  | *Ciz1* | -1.50 |
| *Zfp219* | -1.02 |  | *Ubxn6* | -1.23 |  | *Fbxl21* | -1.52 |
| *9030025P20Rik* | -1.02 |  | *Septin4* | -1.23 |  | *Slc22a17* | -1.52 |
| *Foxq1* | -1.03 |  | *Metap2* | -1.23 |  | *Slc22a18* | -1.52 |
| *Ccdc166* | -1.03 |  | *Gpatch2* | -1.23 |  | *Scd2* | -1.52 |
| *Herc4* | -1.03 |  | *Accs* | -1.24 |  | *Zfp90* | -1.53 |
| *Engase* | -1.03 |  | *Kifc2* | -1.25 |  | *Catsperg1* | -1.53 |
| *Rab11a* | -1.04 |  | *Acat2* | -1.25 |  | *Sstr2* | -1.54 |
| *Stt3a* | -1.04 |  | *Phospho2* | -1.25 |  | *Mmp28* | -1.55 |
| *Washc2* | -1.04 |  | *Dglucy* | -1.25 |  | *Cyth1* | -1.56 |
| *Uckl1* | -1.04 |  | *Ap5z1* | -1.25 |  | *Fn3k* | -1.57 |
| *Spats2l* | -1.05 |  | *Chd2* | -1.25 |  | *Etfbkmt* | -1.57 |
| *Aldh6a1* | -1.05 |  | *Serpinb6a* | -1.26 |  | *Zscan20* | -1.57 |
| *Sirt3* | -1.06 |  | *Prickle3* | -1.27 |  | *Tmed5* | -1.58 |
| *Lman1* | -1.06 |  | *Klhl20* | -1.29 |  | *Fubp3* | -1.58 |
| *Ampd2* | -1.06 |  | *Cpne2* | -1.31 |  | *Aip* | -1.59 |
| *Ssrp1* | -1.06 |  | *Kdm3a* | -1.31 |  | *Acot7* | -1.59 |
| *Ccdc15* | -1.07 |  | *Ctsf* | -1.31 |  | *Cr1l* | -1.60 |
| *Crebl2* | -1.07 |  | *Rfxank* | -1.31 |  | *Tsta3* | -1.61 |
| *Utrn* | -1.07 |  | *Glrx3* | -1.31 |  | *Skap2* | -1.65 |
| *Med11* | -1.08 |  | *Nt5c3* | -1.32 |  | *Pcnt* | -1.67 |
| *Pbld2* | -1.08 |  | *Adhfe1* | -1.32 |  | *Cxcr2* | -1.67 |
| *Stradb* | -1.08 |  | *Prdm9* | -1.33 |  | *Klhl7* | -1.68 |
| *Elmo3* | -1.08 |  | *Wfdc17* | -1.34 |  | *Slc16a5* | -1.72 |
| *Myadm* | -1.09 |  | *Szrd1* | -1.35 |  | *Nxt2* | -1.72 |
| *Tgfb3* | -1.09 |  | *Slbp* | -1.35 |  | *Tepsin* | -1.74 |
| *Zbtb49* | -1.09 |  | *Rabgap1* | -1.35 |  | *Birc5* | -1.76 |
| *Tax1bp3* | -1.10 |  | *Tcirg1* | -1.36 |  | *Gpr137b* | -1.77 |
| *Creg1* | -1.11 |  | *Nrbp2* | -1.36 |  | *Wiz* | -1.78 |
| *Zfp422* | -1.11 |  | *1600012H06Rik* | -1.36 |  | *Dennd4a* | -1.79 |
| *Top3b* | -1.12 |  | *Gnpda2* | -1.39 |  | *Vps33a* | -1.80 |
| *Spg20* | -1.12 |  | *Pcmtd1* | -1.39 |  | *Cyp46a1* | -1.80 |
| *Ppt2* | -1.12 |  | *Mrpl4* | -1.40 |  | *Eml2* | -1.80 |
| *Ankzf1* | -1.12 |  | *S1pr3* | -1.40 |  | *Immp2l* | -1.81 |
| *Rnf13* | -1.13 |  | *Bpgm* | -1.40 |  | *Trf* | -1.82 |
| *Zfp595* | -1.14 |  | *Mcts1* | -1.41 |  | *5730480H06Rik* | -1.82 |
| *Tctex1d2* | -1.14 |  | *Hsf2* | -1.41 |  | *P2rx6* | -1.82 |
| *5330417C22Rik* | -1.14 |  | *Luc7l3* | -1.41 |  | *Hyal3* | -1.84 |
| *Skiv2l* | -1.14 |  | *Lypla1* | -1.43 |  | *Dyrk1b* | -1.85 |
| *Prrg2* | -1.15 |  | *Tom1l2* | -1.43 |  | *Urod* | -1.86 |
| *Rnf38* | -1.15 |  | *Dnajc12* | -1.43 |  | *Yipf4* | -1.89 |
| *Cenpl* | -1.15 |  | *Rasl12* | -1.43 |  | *Tjp2* | -1.89 |
| *Atl2* | -1.15 |  | *Rap1a* | -1.44 |  | *Pfkfb3* | -1.95 |
| *Fbxo6* | -1.15 |  | *Polr3d* | -1.44 |  | *Dctn1* | -1.96 |
| *Upp1* | -1.16 |  | *Hbp1* | -1.45 |  | *Tcp11l2* | -1.97 |
| *Hps4* | -1.16 |  | *Rabggta* | -1.46 |  | *Zfp934* | -1.99 |
| *Rgs3* | -1.18 |  | *Armcx3* | -1.47 |  | *Rabl2* | -2.03 |

| Gene list | Log2fold change |  | Gene list | Log2fold change |  |  |  |
| --- | --- | --- | --- | --- | --- | --- | --- |
| *Kdm2b* | -2.05 |  | *Ggps1* | -4.54 |  |  |  |
| *Sytl4* | -2.05 |  | *Ndufaf1* | -4.57 |  |  |  |
| *Cdkn2d* | -2.06 |  | *Tlk2* | -4.62 |  |  |  |
| *Ptprc* | -2.10 |  | *Skida1* | -4.62 |  |  |  |
| *Klhdc8b* | -2.11 |  | *Tnk1* | -4.90 |  |  |  |
| *Lcp1* | -2.11 |  | *Kcnip2* | -4.91 |  |  |  |
| *Tnni3* | -2.14 |  | *B4galt4* | -4.96 |  |  |  |
| *Klhl10* | -2.17 |  | *Gm16867* | -5.01 |  |  |  |
| *Zfp691* | -2.17 |  | *Uvssa* | -5.08 |  |  |  |
| *Tmem131l* | -2.27 |  | *Pnkp* | -5.14 |  |  |  |
| *Rabep1* | -2.27 |  | *Pip4p1* | -5.20 |  |  |  |
| *Cryzl1* | -2.28 |  | *Pcdhga1* | -5.21 |  |  |  |
| *Pou6f1* | -2.28 |  | *Senp6* | -5.26 |  |  |  |
| *Mpi* | -2.29 |  | *Tmcc2* | -5.38 |  |  |  |
| *Ccdc28a* | -2.31 |  | *1110032A03Rik* | -5.46 |  |  |  |
| *Cyb561a3* | -2.33 |  | *Unk* | -5.46 |  |  |  |
| *Rhot1* | -2.34 |  | *Znrd1as* | -5.59 |  |  |  |
| *Ccl3* | -2.36 |  | *Prc1* | -5.62 |  |  |  |
| *Hacl1* | -2.36 |  | *Capg* | -5.77 |  |  |  |
| *Tmem87b* | -2.37 |  | *Snx8* | -5.81 |  |  |  |
| *Tulp4* | -2.41 |  | *Clp1* | -5.83 |  |  |  |
| *Ankrd16* | -2.41 |  | *Kdm2a* | -5.84 |  |  |  |
| *Plxnb2* | -2.53 |  | *Zfp467* | -6.09 |  |  |  |
| *Ccdc116* | -2.54 |  | *Apbb2* | -6.10 |  |  |  |
| *Ccnd3* | -2.59 |  | *Mtmr12* | -6.11 |  |  |  |
| *Hltf* | -2.60 |  | *Eno3* | -6.31 |  |  |  |
| *Mpc1* | -2.65 |  | *Dock7* | -6.42 |  |  |  |
| *Tmem68* | -2.76 |  | *Gspt1* | -6.50 |  |  |  |
| *Lrmda* | -2.76 |  | *Rsrc2* | -7.99 |  |  |  |
| *Snx21* | -2.79 |  |  |  |  |  |  |
| *Gm45837* | -2.85 |  |  |  |  |  |  |
| *Acaca* | -2.88 |  |  |  |  |  |  |
| *Azin2* | -2.88 |  |  |  |  |  |  |
| *Tia1* | -3.34 |  |  |  |  |  |  |
| *Zfp667* | -3.40 |  |  |  |  |  |  |
| *As3mt* | -3.43 |  |  |  |  |  |  |
| *Zfp446* | -3.57 |  |  |  |  |  |  |
| *Mef2c* | -3.62 |  |  |  |  |  |  |
| *Tbc1d25* | -3.63 |  |  |  |  |  |  |
| *Timm23* | -3.66 |  |  |  |  |  |  |
| *Prpf40b* | -3.78 |  |  |  |  |  |  |
| *Tspo2* | -3.85 |  |  |  |  |  |  |
| *Impact* | -3.97 |  |  |  |  |  |  |
| *Adamts6* | -4.17 |  |  |  |  |  |  |
| *Mfn2* | -4.21 |  |  |  |  |  |  |
| *Mosmo* | -4.21 |  |  |  |  |  |  |
| *Smoc1* | -4.24 |  |  |  |  |  |  |
| *Arhgap27* | -4.30 |  |  |  |  |  |  |
| *4931406C07Rik* | -4.30 |  |  |  |  |  |  |
| *Entpd4* | -4.31 |  |  |  |  |  |  |
| *Lins1* | -4.37 |  |  |  |  |  |  |
| *Hspa2* | -4.39 |  |  |  |  |  |  |
| *Ank3* | -4.43 |  |  |  |  |  |  |
