# Supplementary figures and images for: Loss of Nudt15 thiopurine detoxification increases direct DNA damage in hematopoietic stem cells
Source: Sci Rep. 2023 Jul 24;13:11908. doi: 10.1038/s41598-023-38952-7 (PMC10366091; doi:10.1038/s41598-023-38952-7)

Supplementary Figure 1

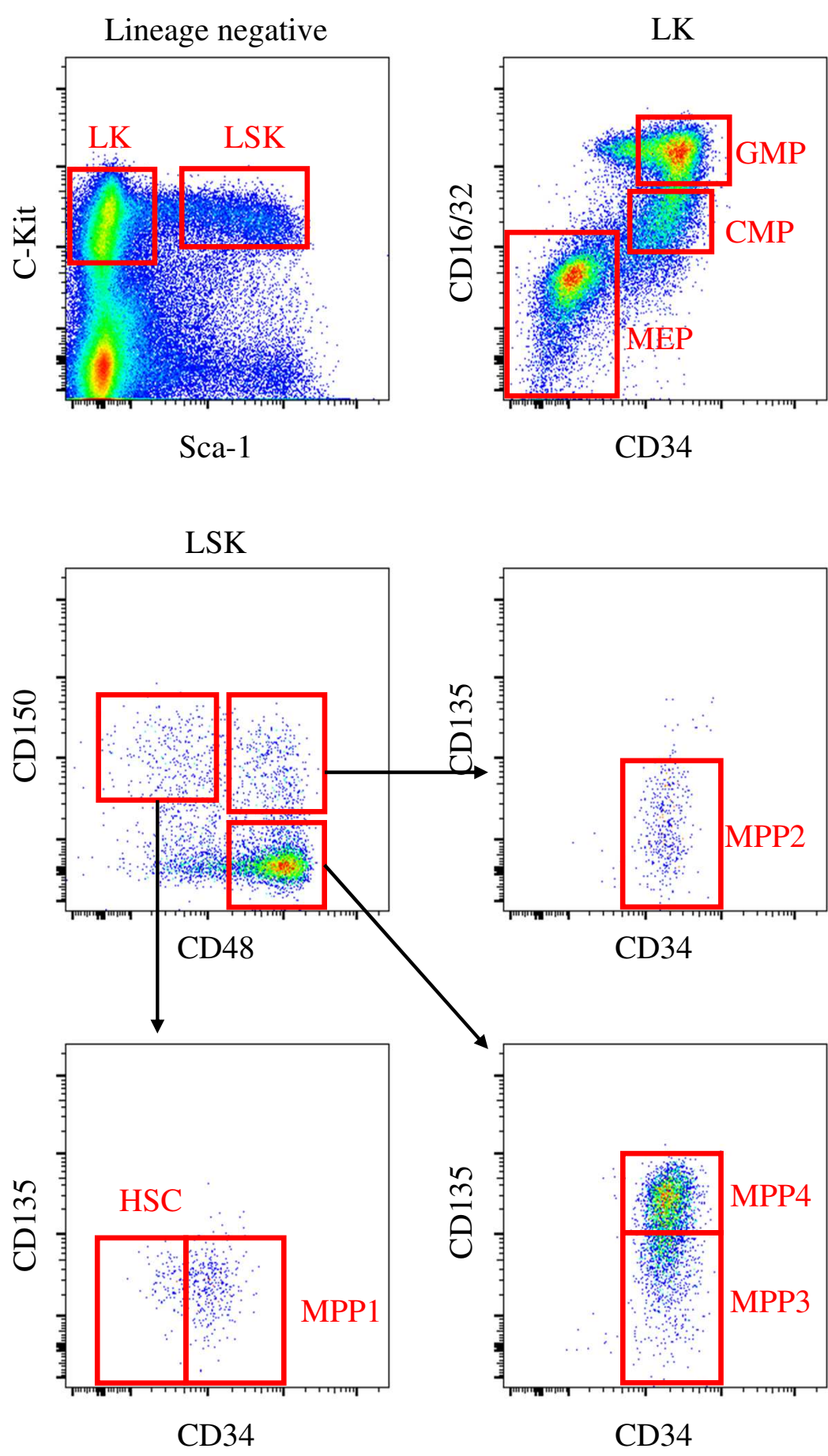

Supplementary Figure 2

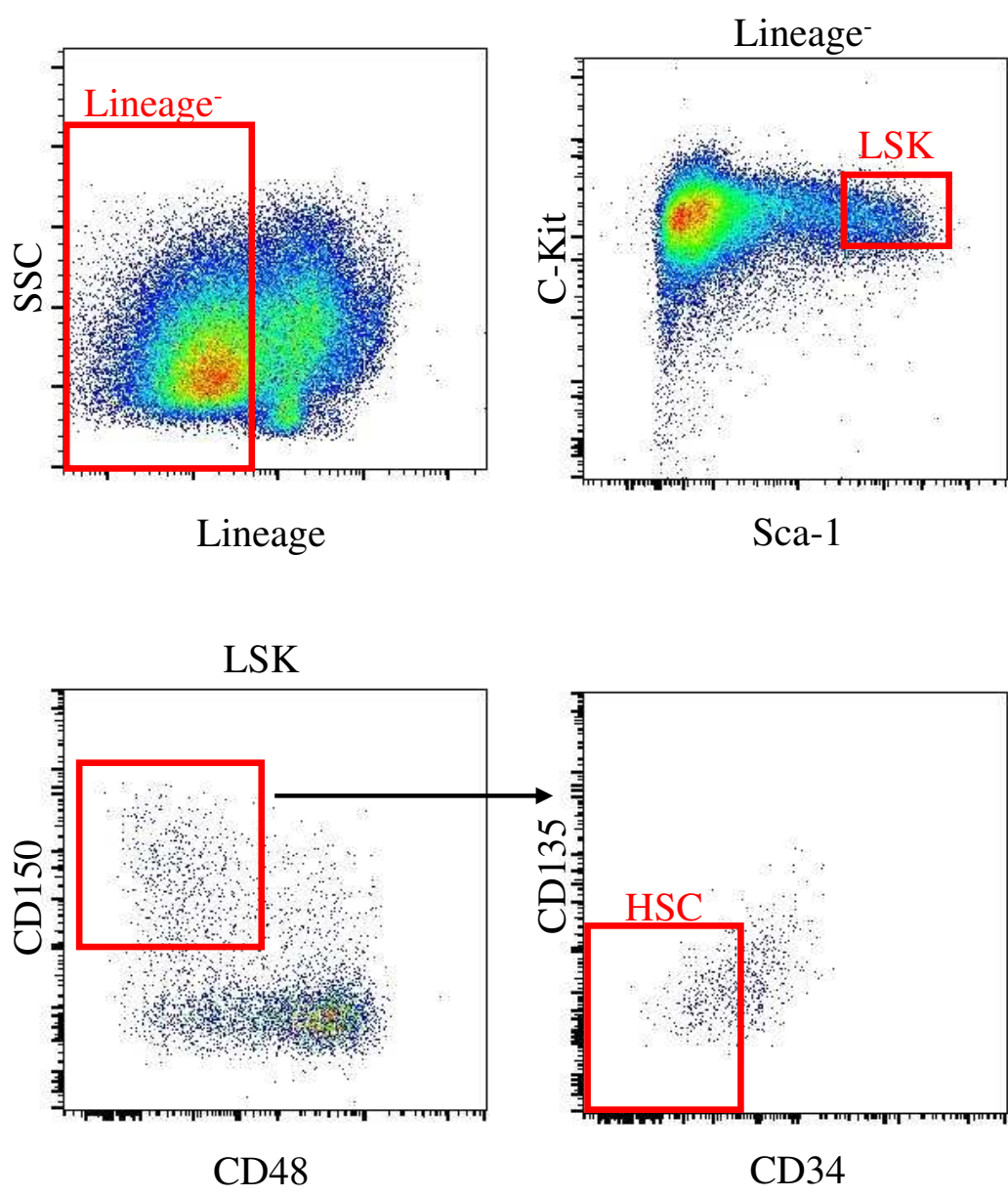

Supplementary Figure 3

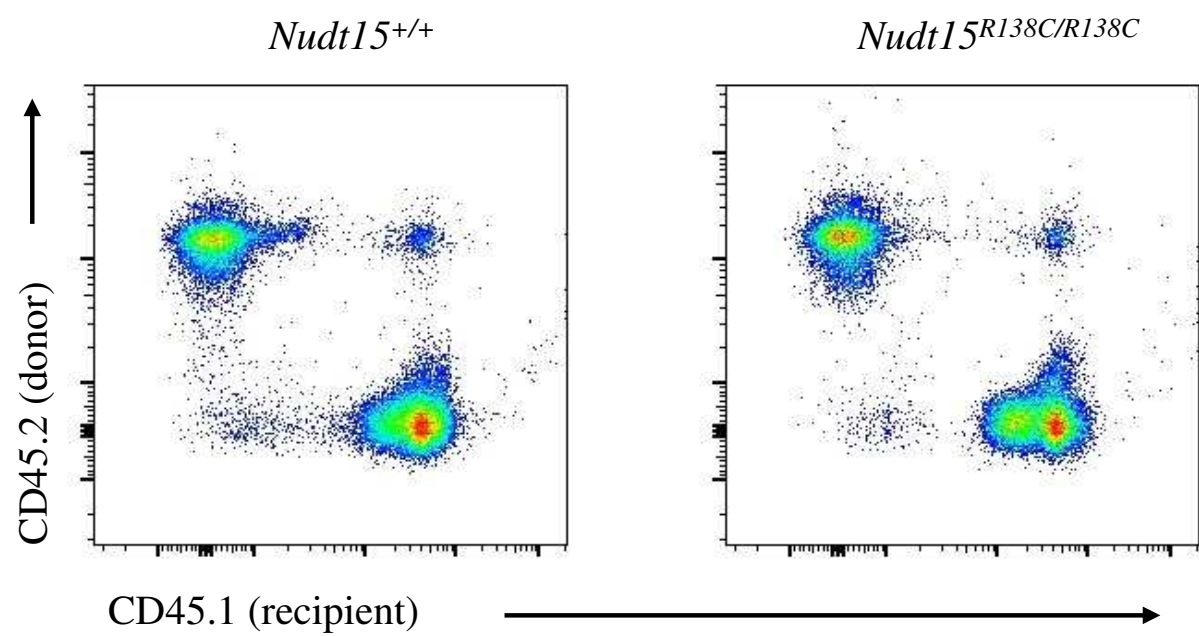

Supplement: Supplementary file 2 — Supplementary Information 2. [file 41598_2023_38952_MOESM2_ESM.pdf]
